# Suppression of phase transitions and glass phase signatures in mixed cation halide perovskites

**DOI:** 10.1038/s41467-020-18938-z

**Published:** 2020-10-09

**Authors:** Mantas Simenas, Sergejus Balciunas, Jacob N. Wilson, Sarunas Svirskas, Martynas Kinka, Andrius Garbaras, Vidmantas Kalendra, Anna Gagor, Daria Szewczyk, Adam Sieradzki, Miroslaw Maczka, Vytautas Samulionis, Aron Walsh, Robertas Grigalaitis, Juras Banys

**Affiliations:** 1 Faculty of Physics, Vilnius University, Sauletekio 3, 10257 Vilnius, Lithuania; 2 Thomas Young Centre and Department of Materials, Imperial College London, SW7 2AZ London, UK; 3 Mass Spectrometry Laboratory, Center for Physical Sciences and Technology, Sauletekio 3, 10257 Vilnius, Lithuania; 4 Institute of Chemical Physics, Vilnius University, Sauletekio 3, 10257 Vilnius, Lithuania; 5 Institute of Low Temperature and Structure Research, Polish Academy of Sciences, Okólna 2, 50-422 Wroclaw, Poland; 6 Department of Experimental Physics, Wroclaw University of Science and Technology, Wybrzeze Wyspianskiego 27, 50-370 Wroclaw, Poland; 7 Department of Materials Science and Engineering, Yonsei University, 03722 Seoul, Korea

**Keywords:** Ferroelectrics and multiferroics, Phase transitions and critical phenomena, Materials for devices, Solar cells

## Abstract

Cation engineering provides a route to control the structure and properties of hybrid halide perovskites, which has resulted in the highest performance solar cells based on mixtures of Cs, methylammonium, and formamidinium. Here, we present a multi-technique experimental and theoretical study of structural phase transitions, structural phases and dipolar dynamics in the mixed methylammonium/dimethylammonium MA_1-*x*_DMA_*x*_PbBr_3_ hybrid perovskites (0 ≤ *x* ≤ 1). Our results demonstrate a significant suppression of the structural phase transitions, enhanced disorder and stabilization of the cubic phase even for a small amount of dimethylammonium cations. As the dimethylammonium concentration approaches the solubility limit in MAPbBr_3_, we observe the disappearance of the structural phase transitions and indications of a glassy dipolar phase. We also reveal a significant tunability of the dielectric permittivity upon mixing of the molecular cations that arises from frustrated electric dipoles.

## Abstract (web summary)

Understanding the underlying physics and improving the performance of mixed lead halide perovskite materials remains a challenge. Here, the authors present experimental and theoretical study of structural phases, phase transitions, and dipolar dynamics in the mixed cation MA1-xDMAxPbBr3 perovskites.

## Introduction

The methylammonium (MA, CH_3_NH_3_^+^) lead halides MAPbX_3_ (X = I, Br, Cl) are extensively investigated perovskite-structured materials for effective and affordable solar cells^1–3^. The power conversion efficiency of cells based on these hybrid compounds has rapidly exceeded more than 25% during the past decade^4–9^. Several key physical factors such as large absorption coefficient^10^, optimal bandgap^11^, long carrier diffusion length^12^, low exciton binding energy^13^, and exceptional defect tolerance^14^ result in a high performance of these materials. However, a further successful application of hybrid perovskite solar cells is hindered by their poor thermal and water stability^15,16^, as well as lead toxicity^17^

The best performance and most stable perovskite solar cells are obtained by employing perovskites with mixed A-site cations^18,19^. The most popular alternatives to MA are organic formamidinium (FA, HC(NH_2_)_2_^+^) and inorganic Cs^+^ ions^20–23^, although less popular cations such as Rb^+^ are also promising^24^. The FAPbI_3_ and CsPbI_3_ compounds crystallize to the photoinactive yellow phases at room temperature, but the desirable photoactive black phases can be stabilized by the mixing of the A-site cations^25–27^. The phase stability and cell performance can be further improved by tuning the halide composition at the X-site^19^.

Recently, dimethylammonium (DMA, (CH_3_)_2_NH_2_^+^) has been proposed as another alternative A-site cation in these compounds^28–31^. Several investigations reveal that during certain synthesis procedures relatively high quantities of DMA may be unintentionally mixed into the structures of MAPbI_3_ and CsPbI_3_^32–34^. These and other studies also indicate that the incorporation of DMA cations can stabilize the desirable cubic phase of MAPbI_3_^28,35^ and the photoactive black phase of CsPbI_3_^30,33,36^ leading to the enhanced performance at room temperature. It is also recognized that such mixing may significantly improve water stability of the solar cells operating at ambient conditions^28,37^. A study by Anelli et al.^31^ has revealed a limited DMA solubility of about 30% in MAPbBr_3_. Note that pure DMA lead halides form two-dimensional hexagonal structures and are unsuitable for photovoltaic applications^30,38–41^.

It is well established for classical inorganic perovskites that mixing may significantly perturb structural phase behavior^42^. In this way the long-range order can be suppressed and frustrated phases such as relaxor^42,43^ or dipolar glass^44,45^ may emerge. Dielectric anomalies associated with these phases are usually very broad with respect to both frequency and temperature. In addition, at sufficiently low temperature, a freezing of the electric dipoles may occur with dynamics described by the Vogel–Fulcher law instead of the typical Arrhenius behavior^43,46,47^.

The dielectric properties of lead halides seem to be especially important for the performance of the perovskite solar cells, as their relatively high value of the dielectric permittivity results in a pronounced defect tolerance and low exciton binding energy^48–50^. In addition to solar cells, other devices such as memristors based on these materials also highly rely on their dielectric response^51,52^. However, despite significant literature focused on improving the performance of mixed lead halide perovskite materials, a comprehensive understanding of mixing effects on the dipolar dynamics, dielectric properties, and structural phase behavior is still absent. Here, we present a joint experimental and theoretical study of these phenomena for the mixed hybrid perovskite MA_1−x_DMA_x_PbBr_3_. Our choice of the DMA cation is motivated by its relatively high permanent electric dipole moment and the rich dielectric properties of the related DMA metal-formate frameworks^53,54^. The MAPbBr_3_ analog was selected due to its cubic crystal symmetry at room temperature, well-spread phase transitions and less demanding synthesis. Our results indicate that even a low concentration of the DMA cations significantly suppresses the structural phase transitions. For higher DMA levels, we observe signatures that suggest the frustration of electric dipoles, which both disrupts long-range dipolar order and potentially induces the formation of a dipolar glass phase.

## Results

### Initial sample characterization

Our study was carried out on the mixed MA_1−x_DMA_x_PbBr_3_ perovskite crystals with *x* = 0.04, 0.14, and 0.21. For comparison, crystals of MAPbBr_3_ and DMAPbBr_3_ compounds were also investigated. The concentration of the DMA cations was determined by measuring the atomic *C*/*N* ratio (see “Methods” section). Crystals with intermediate DMA concentrations (0.21 < *x* < 1) were not synthesized due to the finite DMA solubility of about 30% in MAPbBr_3_, as has been reported previously^31^. The initial powder X-ray diffraction (XRD), Raman spectroscopy, and light absorption characterization of the samples proved the successful incorporation of the DMA cations into the crystal lattice (see Supplementary Figs. 2–4).

### Differential scanning calorimetry (DSC) experiments

**Fig. 1:**
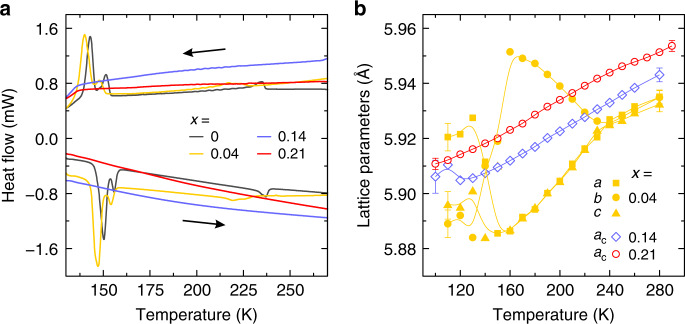
Phase transition behavior of mixed MA_1−x_DMA_x_PbBr_3_ perovskites. **a** DSC traces of *x* = 0, 0.04, 0.14 and 0.21 compounds obtained on cooling and heating. **b** Temperature dependence of the crystal lattice parameters of *x* = 0.04, 0.14 and 0.21 compounds determined by the single crystal XRD. The curves in **b** are guides for eyes.

The DSC measurements of the mixed compounds are presented in Fig. 1a. In agreement with previous studies, three structural phase transitions are clearly observed at 236, 153, and 144 K on cooling for MAPbBr_3_, corresponding to the cubic → tetragonal-I → tetragonal-II → orthorhombic transitions, respectively^48,55^. For the *x* = 0.04 system, the DSC anomalies associated with the phase transitions become significantly broader and occur at lower temperatures. The most pronounced shift (of about 15 K) is observed for the cubic to tetragonal-I phase transition, indicating that even a small amount of DMA helps to stabilize the cubic phase. Other transition temperatures are lowered by about 4 K. For higher DMA concentration, the phase transition anomalies are not resolved suggesting significant disruption of the long-range ordering. We observe a single structural phase transition at 250 K on cooling for the hexagonal DMAPbBr_3_ compound (*x* = 1) (see Supplementary Fig. 5) in agreement with a previous study^41^.

### Single crystal XRD experiments

The results of the single crystal XRD measurements clearly reveal anomalous behavior for *x* = 0 (Supplementary Fig. 6) and 0.04 compounds (Fig. 1b). The phase transitions occur at lower temperatures for the *x* = 0.04 material, consistent with the DSC results. This effect is further enhanced for the *x* = 0.14 compound, such that a deviation from the cubic symmetry appears only below 130 K (Fig. 1b). The complex diffraction picture of the low temperature phase of this compound is similar to the orthorhombic phase observed for *x* = 0 and 0.04. This implies that the intermediate tetragonal phases have been suppressed. Following this trend, a suppression of all structural phase transitions is observed for the *x* = 0.21 compound (Fig. 1b). The diffraction picture is consistent throughout the temperature range investigated, as the crystal remains cubic. Reciprocal space reconstructions can be seen at selected temperatures in Supplementary Figs. 7, 8.

### Ultrasonic velocity and attenuation experiments

**Fig. 2:**
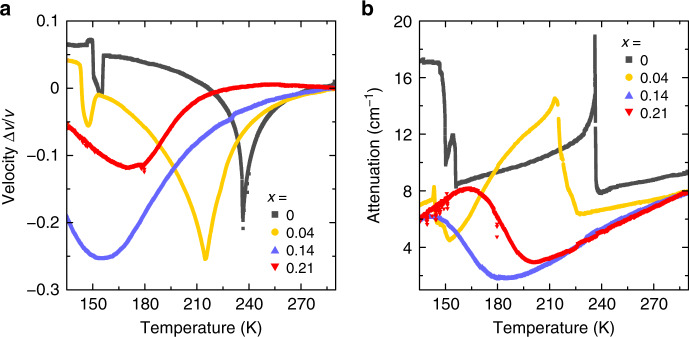
Ultrasonic properties of mixed MA_1−x_DMA_x_PbBr_3_ perovskites. **a** Ultrasonic velocity and **b** attenuation of the *x* = 0, 0.04, 0.14, and 0.21 single crystal compounds measured on cooling. The change of the ultrasonic velocity is normalized to the room temperature value.

We further studied the mixed compounds by performing ultrasonic velocity and attenuation measurements of single crystal samples. Such experiments proved to be highly powerful in detecting and characterizing the phase transitions in lead halides perovskites^48^. The ultrasonic velocity and attenuation measurements detect anomalies at the expected phase transition points for a pure MAPbBr_3_ single crystal (Fig. 2). The anomalies are significantly shifted and broadened upon introduction of a small amount of DMA (*x* = 0.04) in agreement with the DSC and XRD data. Further increase of the DMA content results in a clear suppression of the phase transitions, as only a single and very broad anomaly is observed below 200 K. It is noteworthy that similar ultrasonic behavior is frequently observed in frustrated (disordered) electric dipole systems such as relaxors or dipolar glasses^56,57^.

### Broadband dielectric spectroscopy experiments

**Fig. 3:**
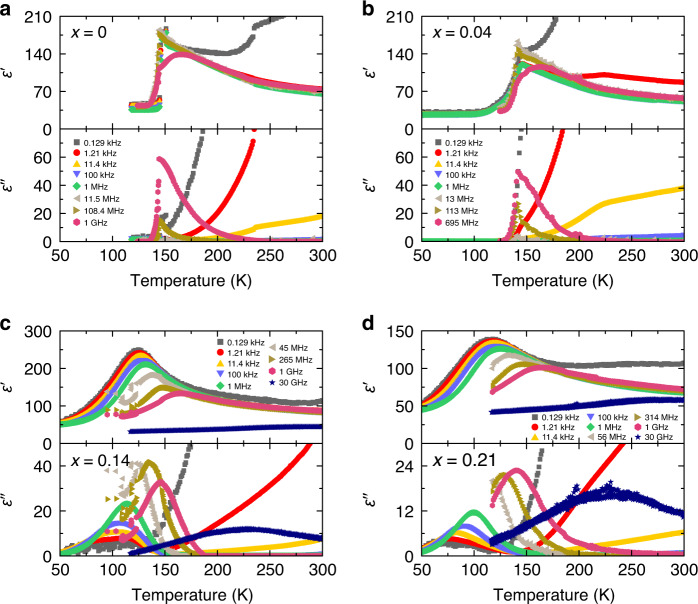
Dielectric behavior of mixed MA_1−x_DMA_x_PbBr_3_ perovskites. Temperature dependence of the real and imaginary parts of the complex dielectric permittivity at selected frequencies of **a** *x* = 0, **b** 0.04, **c** 0.14, and **d** 0.21 single crystal compounds measured on cooling.

We further used broadband dielectric spectroscopy to probe the phase transitions and dipolar dynamics in the extensive frequency range from 20 Hz to 50 GHz. The obtained temperature dependences of the real *ε*′ and imaginary *ε*′′ parts of the complex dielectric permittivity ε* = *ε*′ − *iε*′′ are presented in Fig. 3 for *x* = 0, 0.04, 0.14 and 0.21 compounds. For MAPbBr_3_ (Fig. 3a), an anomalous behavior of ε* is clearly observed at the tetragonal-II → orthorhombic phase transition point in agreement with previous studies^48,50^. A sharp decrease of ε* indicates a first-order structural phase transition and can be related to the (antipolar) ordering of the MA cations in the orthorhombic phase. The dielectric anomalies associated with other phase transitions are significantly less pronounced. The dielectric permittivity data for DMAPbBr_3_ (*x* = 1, Supplementary Fig. 9) also reveal a first-order structural phase transition in agreement with the DSC results and previous investigation^41^.

For sample containing a 4% DMA concentration (Fig. 3b), the temperature behavior of the dielectric permittivity is very similar to MAPbBr_3_. However, the anomalous decrease of ε* is more gradual and occurs at lower temperature in agreement with other measurements. The situation drastically changes upon further increase of the DMA content (*x* = 0.14) as revealed in Fig. 3c. For this compound, no structural phase transitions can be identified down to 50 K. Instead, the temperature dependence of ε* is dominated by a very broad and highly frequency-dependent anomaly. The anomaly is significantly broader and occurs at lower temperature for the material containing 21% of DMA cations (Fig. 3d).

**Fig. 4:**
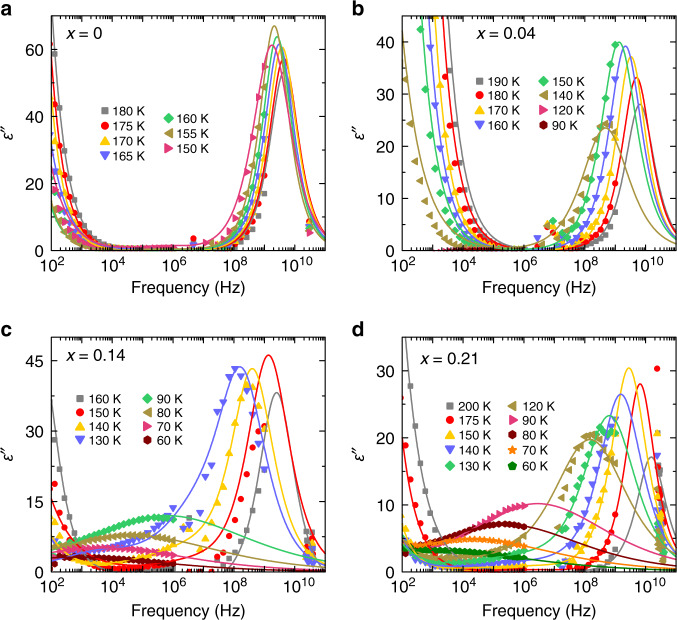
Frequency dependent dielectric permittivity of mixed MA_1−x_DMA_x_PbBr_3_ perovskites. Frequency dependence of the imaginary part of the complex dielectric permittivity at selected temperatures of **a** *x* = 0, **b** 0.04, **c** 0.14, and **d** 0.21 single crystal compounds. The curves are the best fits to Eq. (1).

A comparison with classical inorganic compounds suggests that such broad dielectric anomalies may occur due to the frustration of electric dipoles and the emergence of a glassy phase^42,43,45^. Further indications of the glassy behavior of our compounds are revealed by analyzing the complex dielectric permittivity in the frequency domain (Fig. 4 and Supplementary Fig. 10). For the *x* = 0 and 0.04 compounds, a strong relaxation process related to the MA cation dynamics^49^ can be identified in the GHz frequency range. This process is terminated by the tetragonal-II → orthorhombic phase transition, as the long-range cation order is established. However, for higher DMA mixing levels, the relaxation still persists to the lowest temperature accessible by our equipment and extends into the low frequency domain. The additional low-temperature experiments covering the mHz frequency range performed on *x* = 0.21 compound reveal a very broad relaxation, which extends to even lower frequencies (Supplementary Fig. 11). These results indicate a gradual slowing down of the dipolar dynamics by approximately twelve orders of magnitude and imply formation of a glassy disordered phase. Further support for the emergence of a glassy phase is provided by the evolution of the dielectric curves throughout the frequency and temperature range investigated. Such behavior is typically observed for other dipolar glasses^45,46,58^. Note that the increase of ε* observed at low frequencies for all compounds is not related to the molecular dynamics, but instead occurs due to the conductivity effects^59^.

To further investigate the existence of the dipolar glass phase, the frequency dependences of ε* were approximated by the superposition of two Cole–Cole processes that describes both cation relaxation and conductivity effects^59^:

1 $$\varepsilon ^ {*} \left( \omega \right)\,=\,\varepsilon \left( \infty \right) + \mathop {\sum}\limits_j {\frac{{{\Delta}\varepsilon _{{j}}}}{{1 + ( {i\omega \tau _{{j}}} )^{1 - \alpha _{{j}}}}}}.$$

Here *ε*(∞) is the dielectric permittivity in the high-frequency limit, and *ω* = 2π*ν* is the angular measurement frequency. Index *j* enumerates each process described by a dielectric strength Δ*ε*_*j*_, a mean relaxation time *τ*_*j*_ and a relaxation width 0 ≤ *α*_*j*_ < 1. For *α*_*j*_ = 0, the Cole–Cole process reduces to the Debye relaxation equation, which describes noninteracting electric dipoles^59^. The best fits of Eq. (1) to the frequency domain data are also presented in Fig. 4 and Supplementary Fig. 10. For *x* = 0 and 0.04 compounds, the obtained value of *α* describing the cation relaxation is approximately zero for the entire temperature range investigated. For higher concentrations, *α* gradually increases from 0 to a value close to 1 upon cooling. Such behavior is typical of a dipolar glass^58^. The value of *ε*(∞) varies from about 25–35 for low and high DMA concentrations, respectively, as is expected for these materials^50^.

The obtained temperature dependence of the mean relaxation time for the dominant dipolar process is presented in Supplementary Fig. 12. For all samples it follows the Arrhenius law: *τ* = *τ*_0_exp(*E*_a_/*kT*), where *E*_a_ and *τ*_0_ denote the activation energy and attempt time, respectively, and *k* is the Boltzmann constant. The determined activation energies are 80(5), 113(7), 143(7), and 140(7) meV for *x* = 0, 0.04, 0.14, and 0.21, respectively. The corresponding values of *τ*_0_ are 1.8(3) × 10^−13^, 2.1(2) × 10^−14^, 2.9(1) × 10^−15^, and 1.2(1) × 10^−15^ s. The absence of a Vogel–Fulcher behavior of *τ* for the *x* = 0.14 and 0.21 compounds suggests that the freezing of electric dipoles might occur at temperatures below 50 K.

### Heat capacity measurements

To further investigate this, we also performed low temperature measurements of the heat capacity *C*_p_ of *x* = 0, 0.14, 0.21, and 1 compounds (Supplementary Fig. 13). A typical signature of a glassy phase is a higher value of the heat capacity and its deviation from the expected temperature dependence, which is best revealed as a maximum in the *C*_p_/*T*^3^ vs. *T* representation^45^. Indeed, our experiments indicate such a maximum slightly below 10 K, although it is also observed for the non-mixed compounds. However, another clear increase of *C*_p_/*T*^3^ can be seen below 3 K only for the mixed systems (*x* = 0.14 and 0.21) indicating a higher entropy state compared to the phase pure compounds. Such a behavior might originate from the glassy phase. We note that such a low dipolar glass freezing temperature is typical for various dipolar glass systems^45^. The origin of the *C*_p_/*T*^3^ maximum observed for all compounds might be tentatively assigned to defect states, although its precise origin is beyond the scope of this study.

### Density functional theory (DFT) calculations

**Fig. 5:**
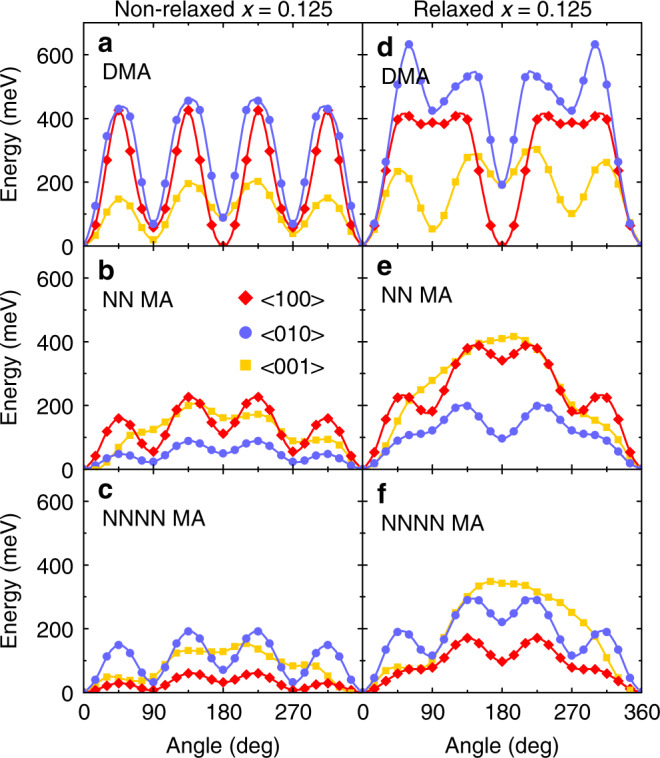
The potential energy surfaces obtained by rotating DMA and neighboring MA cations within a MA_0.875_DMA_0.125_PbBr_3_ system. Rotations of **a**, **d** DMA and **b**, **c**, **e**, **f** MA cations are performed in **a**–**c** nonrelaxed and **d**–**f** relaxed structures of MA_0.875_DMA_0.125_PbBr_3_ system. The cations are rotated around the <100> (red), <010> (blue) and <001> (yellow) axes. The rotation axes cross the cation center of mass. The curves are guides for eyes.

To support our experimental findings, we performed DFT calculations on a mixed MA_0.875_DMA_0.125_PbBr_3_ supercell (see Supplementary Fig. 14 and Supplementary Note 3 for more details). A potential energy surface was constructed by rotating the DMA and two neighboring (nearest neighbor (NN) and next-next-nearest neighbor (NNNN)) MA cations around three orthogonal lattice directions (see Fig. 5d–f). A comparison between the rotational dynamics of molecular cations within the cavity of the relaxed, pseudo-tetragonal structure and the nonrelaxed, pseudo-cubic structure was made (Fig. 5a–c). The cation rotation potentials are clearly different in both structures demonstrating that the neighboring MA cations are significantly perturbed by the presence of a bigger DMA cation. Rather than arising from dipole–dipole interactions, our results suggest that the perturbation is facilitated via the observed lattice deformation, which is induced by the DMA cation (also see Supplementary Fig. 14). In addition, the average barrier of MA rotation decreases with increasing distance to the DMA cation reflecting that the lattice deformation is a local effect (Fig. 5e, f). Our calculations also show that the average barrier of DMA rotation is substantially higher compared with the distant MA cations resulting in much slower dynamics of DMA. In addition to a much lower amount of DMA, this allows us to conclude that the observed dielectric response of the mixed compounds originates from the MA cations. More importantly, in the mixed compounds, a random distribution of DMA causes different degree of framework distortion, which results in a multiwell potential (competing interactions) for MA cations causing frustration and suppression of the phase transitions.

### Monte Carlo simulations

**Fig. 6:**
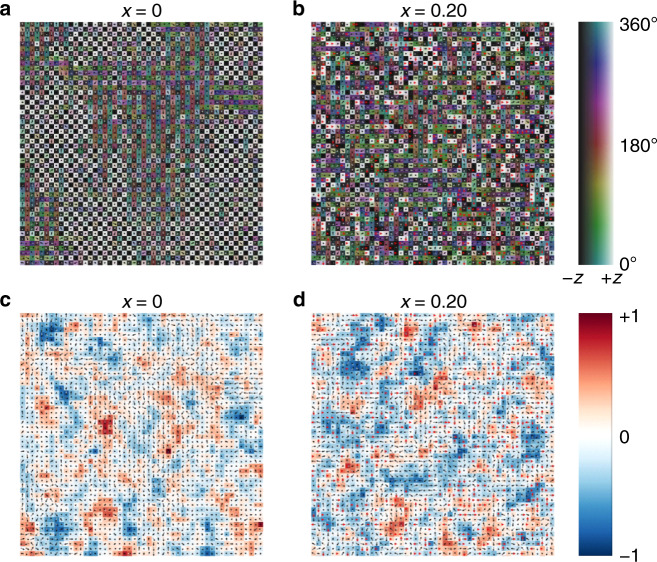
Monte Carlo simulations of mixed MA_1−x_DMA_x_PbBr_3_ perovskites. **a**, **b** Snapshots of Monte Carlo simulations and **c**, **d** potential energy landscape for a two-dimensional slice of a three-dimensional periodic slab representing MA_1-*x*_DMA_*x*_PbBr_3_ for **a**, **c** *x* = 0, and **b**, **d** *x* = 0.20. The orientations of the MA and DMA dipoles are represented by gray and red arrowheads, respectively. The MA dipole is allowed to freely rotate, however, the orientation of the DMA dipole is fixed toward a <100> (or equivalent) lattice direction. The color bar in **a**, **b** represents the angle of the dipole with respect to the <100> direction; it is darkened towards <00-1> (−z) and lightened towards <001> (+z). The color bar in **c**, **d** shows the normalized electrostatic potential.

We further performed Monte Carlo simulations on the mixed compounds to investigate the impact of DMA cations, which we treated as rotationally inactive, on the long-range order of MA (see “Methods” section for simulation details). The employed model treats the molecular cation system as interacting point dipoles^60^. Note that for simplicity, we have not considered interactions mediated by the framework deformation that proved to be important for obtaining the correct phase transition sequence in MAPbX_3_^61,62^. In order to model the rotationally inactive DMA cation, simulations were performed by randomly fixing orientations of the DMA dipoles along <100> (or equivalent) lattice directions. As can be seen in Fig. 6a, c, the *x* = 0 system converges into domains of antipolar checkerboard configuration as is expected for the orthorhombic phase of MAPbBr_3_. A similar behavior can be observed for the *x* = 0.04 system (Supplementary Fig. 15a, c). However, an increase of DMA to *x* = 0.14 introduces significant disorder such that distinct domains are no longer visible (Supplementary Fig. 15b, d). A similar situation emerges for the *x* = 0.20 system (Fig. 6b, d), which shows a complete lack of the long-range dipole order. The larger fluctuations in the potential energy landscape seen in Fig. 6d suggest that the MA dipoles experience more severe competing interactions due to the frozen DMA cations.

## Discussion

**Fig. 7:**
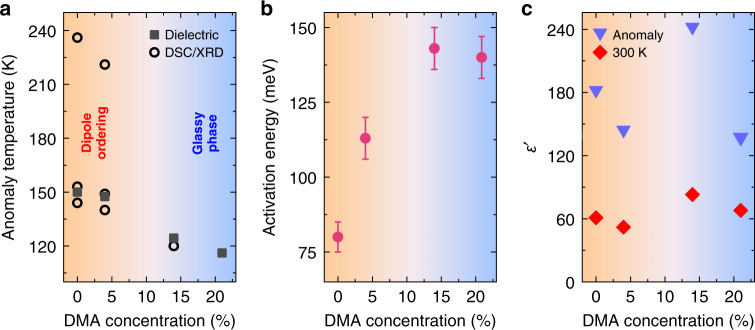
Summary of experimental results. DMA concentration dependence of **a** phase transition and dielectric anomaly temperatures, **b** activation energy of molecular cation motion and **c** dielectric permittivity of the anomaly (129 Hz) and at 300 K (1 MHz). The bluish part of the phase diagram marks the glassy behavior of the electric dipoles. If not indicated, the error bars are smaller than the data points.

We summarized some of our experimental results in the phase diagram depicted in Fig. 7. The introduction of the DMA cations in the crystal significantly shifts the dielectric anomaly to lower temperatures and suppresses the phase transitions (Fig. 7a). The obtained increase of the activation energies with the DMA concentration indicates a gradual rise of the rotational barrier for the MA cation motion (Fig. 7b). The dielectric permittivity at room temperature and its value at the dielectric anomaly are highest for the *x* = 0.14 compound, which is slightly less frustrated than *x* = 0.21 (Fig. 7c). This demonstrates that the room temperature dielectric permittivity of hybrid perovskites can be significantly tuned (up to 50%) by mixing of the A-site cations.

We also note that the vast broadening of the dielectric relaxation of the mixed systems prevented us to investigate the relaxation time at low temperature and thus to provide a strong evidence (in the form of the Vogel–Fulcher freezing) of the existence of the dipolar glass phase. Whilst this is not conclusive, support for the occurrence of this phase in the mixed compounds is provided by our experimental and theoretical data. Namely, a comparison with known dipolar glass systems reveals that the temperature dependences of the ultrasonic, heat capacitance and dielectric properties of the *x* = 0.14 and 0.21 compound are typical for the glassy phase. Moreover, the DFT calculations show that due to induced lattice distortion, the MA cations exhibit a complicated potential energy surface likely resulting in competing interaction picture, which is a prerequisite of frustration and glass phase formation.

In addition, our dielectric data suggest a dipolar glass phase rather than the relaxor state, which is more typical for the displacive compounds^43,47^. Canonical relaxors such as PbMg_1/3_Nb_2/3_O_3_ exhibit a constant dielectric loss regime as a freezing temperature is approached, where the dielectric losses are independent of frequency^43^. In contrast, our low-frequency dielectric experiments performed at low temperature indicate that the dielectric losses are frequency dependent (Fig. 4 and Supplementary Fig. 11). In addition, relaxor ferroelectrics typically exhibit multi-component dielectric relaxation^63^. In contrast, a single process is sufficient to describe the dielectric relaxation of the investigated samples. The only other observed contribution stems from the conductivity.

It is interesting to note that there are some indications in the literature of the dipolar glass behavior at low temperature associated with the dielectric properties of MAPbBr_3_, MAPbI_3_, and FAPbI_3_ lead halides^64,65^. However, the reported magnitudes of the dielectric dispersions are much weaker in comparison to our results. This may indicate that for the single cation systems, point or extended defects could act as a source of local frustration (lattice deformation) for the molecular cations, whilst in our case, the frustration percolates the whole crystal. Suppression of the long-range order by frustration upon mixing may also explain the stabilization of the black phases of FAPbI_3_ and CsPbI_3_.

In conclusion, we report a comprehensive study of the structural phase transitions and molecular cation dynamics in the mixed MA_1−x_DMA_x_PbBr_3_ perovskites. Even for a low amount of DMA (*x* = 0.04), the anomalies of the structural phase transitions are broadened and occur at noticeably lower temperatures, although the long-range molecular cation order seems to be preserved. The intermediate DMA concentration level (*x* = 0.14) results in the significant promotion of the cubic phase and complete suppression the tetragonal phases. For the highest investigated DMA concentration (*x* = 0.21), the system remains in the cubic phase due to the frustrated interactions between the molecular cations caused by lattice deformation. This likely leads to the formation of the glassy phase of electric dipoles, although a strong evidence of such a behavior seems to be evasive in this system. Our results also demonstrate a significant tunability of the dielectric permittivity by molecular cation mixing.

All these aspects are highly important for understanding and improving the performance and stability of the photoactive phases of perovskite solar cells and associated technologies. We anticipate that the observed phase transition suppression and indications of the glassy phase might be more general in the mixed compounds and could be invoked using different mixing recipes at all three lattice sites of the hybrid perovskites. It would be especially interesting to investigate compounds, where mixing is not limited by the solubility limit and thus much broader range of concentration can be covered (e.g., mixed MA/formamidinium perovskites).

## Methods

### Sample preparation

Single crystals of MA_1−x_DMA_x_PbBr_3_ were grown using antisolvent vapor-assisted crystallization, in which the appropriate antisolvent is slowly diffused into a solution containing the crystal precursors. In the typical synthesis, 2 mmol of hydrobromic acid was added to appropriate amounts of methanol solutions containing methylamine and dimethylamine (2 mmol). Then 3 mL of dimethyl sulfoxide (DMSO), 5 mL of acetonitrile and 2 mmol of PbBr_2_ were added to the prepared amine hydrobromide solution under stirring. The clear solution obtained after 1 h was transferred into a glass vial and this vial was placed in a second larger glass vial containing methyl acetate. The lid of the outer vial was thoroughly sealed, but the lid of the inner vial was loosened to allow diffusion of the methyl acetate into the precursor solution. Orange crystals were harvested after 5 days, washed with acetonitrile and dried at room temperature (see Supplementary Fig. 1).

### Determination of the DMA concentration

The concentration of DMA cations was determined by measuring the atomic *C*/*N* ratio using Thermo FlashEA 1112 elemental analyzer (EA). It consists of an autosampler (MAS200R), oxidation, and reduction columns, a water trap, a gas chromatographic separation column (PoraPlot Q 6m) and a thermal conductivity (TCD) detector. Eager300 software was used to operate the EA system. During the measurements, the EA was permanently flushed with He gas. The water trap was filled with magnesium perchlorate. At 24 s after the start of the experiment, the autosampler loaded a sample (weighted and packed in a tin capsule) into the oxidation reactor, and oxygen was subsequently injected into the reactor. The oxidation reactor was a packed quartz tube filled with chromium(III) oxide and granular silvered cobalt oxide in a top-to-down sequence. The sample was oxidized to CO_2_ and nitric oxides (NO_X_) in the presence of excess O_2_ by flash combustion. Subsequently, the NO_X_ was converted into N_2_ gas by reduction using copper. The mixture of gases was passed through a water trap to remove H_2_O. TCD was used to measure the signal intensity of each evolved gas. For the C and N amount determination in the sample, a calibration curve was created using a Nicotinamide standard. Laboratory balances (Mettler Toledo, model XP105) were used for sample weighing. The determination error of the MA/DMA ratio is less than 2%.

### DSC measurements

The powder samples of 46.42 (*x* = 0), 48.15 (*x* = 0.04), 57.37 (*x* = 0.14), and 50.99 mg (*x* = 0.21) mass were packed into a 40 μL aluminum pan. The heat flow was measured using a high resolution (0.04 μW) Mettler Toledo DSC-1 calorimeter. The measurements were performed during heating and cooling at a rate of 5 K/min. The flow rate of nitrogen gas was kept at 30 mL/min. Before the measurements, the calorimeter was calibrated to ensure temperature accuracy of ±0.2 K.

### Heat capacity measurements

Heat capacity at constant pressure *C*_p_ of *x* = 0, 0.14, 0.21 and one samples was measured in the temperature range 1.8–275 K using thermal relaxation technique in the heat capacity option of the physical property measurements system (PPMS). The typical accuracy of the system is better than 1% for temperatures above 100 K and slightly diminishes at lower temperatures. For the measurements, the monocrystals of MAPbBr_3_ and DMAPbBr_3_ were used (8.6 and 4.6 mg, respectively), while mixed compounds were pressed from powders into pellets with a thickness of about 1 mm and weight of 6 mg.

### XRD measurements

Single crystal XRD data were collected on Xcalibur Atlas diffractometer operating with Mo K-α radiation and Oxford Diffraction low-temperature attachment. The lattice parameters were calculated based on the complete data set for the cubic symmetry in CrysAlisPro (CrysAlis PRO 1.171.38.43 (Rigaku OD, 2015)) from room temperature down to 100 K. Lattice parameters for the *x* = 0 and 0.04 samples were calculated with the orthorhombic restraints to present structure distortion in each direction. The data for the *x* = 0.14 and 0.21 samples were treated with the cubic restraints, since the symmetry lowering was not observed for the *x* = 0.21 sample in the entire measured temperature range and down to 120 K in *x* = 0.14. Powder XRD measurements were performed on an X’Pert PRO powder diffractometer operating with Cu K-α radiation. The diffractograms were collected in the Bragg–Brentano geometry using fixed-divergence slits.

### Light absorption experiments

Diffuse reflectance spectra of the powdered samples were measured using the Varian Cary 5E UV–VIS–NIR spectrophotometer.

### Raman spectroscopy

Room temperature Raman spectra were measured using a Bruker FT 100/S spectrometer with YAG:Nd laser excitation (1064 nm). The spectral resolution was set to 2 cm^−1^.

### Ultrasonic experiments

Ultrasonic velocity and attenuation of the longitudinal waves were measured by employing the ultrasonic pulse time-of-flight transmission technique at 10 MHz frequency using Ritec RAM-5000-SNAP system. Temperature dependences of the received signal amplitude and time-of-flight were obtained on heating and cooling at a rate of 0.3 K/min in the vicinity of the anomalies and 1 K/min elsewhere. We present the change of the normalized ultrasonic velocity Δ*v*/*v*, as rather low thickness of the single crystals (about 2 mm) resulted in a relatively high uncertainty (about 5%) of the absolute value.

### Dielectric spectroscopy

Broadband dielectric spectroscopy experiments were performed in four different frequency bands. (i) 10 mHz–100 kHz band: measurements of capacitance and loss tangent with Solartron Modulab XM MTS system together with XM MFA low-current module. (ii) 20 Hz–1 MHz band: measurements of capacitance and loss tangent with HP4284A LCR meter. For both bands, the flat capacitor model was implemented to calculate the complex dielectric permittivity. (iii) 1 MHz–1 GHz band: the complex reflection coefficient was measured with Agilent 8714ET vector network analyzer. The multimode capacitor model was used to calculate the real and imaginary parts of complex dielectric permittivity^66^. (iv) 26–50 GHz band: the scalar transmission and reflection coefficients were measured in a waveguide system. The sample was perpendicularly centered on a wider wall of the waveguide. The dimensions of the waveguide were chosen such that only the fundamental H_10_ mode can propagate in it. Transmission/reflection coefficients were measured with a scalar network analyzer Elmika R2400. A detailed description of the experimental setup can be found in literature^67^. Temperature dependent dielectric spectra were measured on cooling at a rate of 1 K/min. Temperature was measured with a Keithley Integra 2700 multimeter, a T-type thermocouple and a 100 Ω platinum resistor. For measurements at low frequency (10 mHz–100 kHz band), the temperature was stabilized before performing the experiment.

### DFT calculations

The MA_1−x_DMA_x_PbBr_3_ mixed perovskite structures and energies were calculated using DFT methods (employing the ab initio code VASP^68^). A 2 × 2 × 2 supercell was generated in which seven of the A-site atomic positions were occupied by MA cations and one by a DMA. Calculations were performed with a plane wave cutoff energy of 700 eV; a 3 × 3 × 3 Monkhurst-Pack k-point sampling grid at the gamma point, PAW pseudopotentials^69^, and the PBEsol exchange-correlation functional^70^.

### Monte Carlo simulations

The open-source Monte Carlo code, StarryNight, was implemented for all simulations^60^. In the used model, the molecular cations are treated as point dipoles interacting only via the dipolar interactions. Literature values of 2.29 D and 0.73 D were utilized for the dipole moment of the MA and DMA molecules, respectively^11,71^. Each dipole site underwent an average of 8000 Monte Carlo moves before data collection. The source code was modified to allow selective dynamics for the MA and DMA molecules. Simulations were performed at 100 K on a 100 × 100 × 100 (≈ 1.25 × 105 nm^3^) representative MA_1−x_DMA_x_PbBr_3_ system for *x* = 0, 0.04, 0.14, and 0.20. Simulations were initiated with a random cation configuration. More details about the interaction Hamiltonian and computational information can be found in ref. ^60^.

## Supplementary information

Supplementary Information

## Data Availability

The data that support the findings of this work are available from the corresponding author on request.

## Code availability

The MC code is available at https://github.com/WMD-group/StarryNight.
