# Supplementary material for: Suppression of phase transitions and glass phase signatures in mixed cation halide perovskites
Source: Nat Commun. 2020 Oct 9;11:5103. doi: 10.1038/s41467-020-18938-z (PMC7547736; doi:10.1038/s41467-020-18938-z)
Supplement: Supplementary file 1 — Supplementary Information [file 41467_2020_18938_MOESM1_ESM.pdf]

Supplementary Information

Suppression of phase transitions and glass phase signatures in mixed cation halide perovskites

Mantas Simenas,* Sergejus Balciunas, Jacob N. Wilson, Sarunas Svirskas, Martynas Kinka, Andrius Garbaras, Vidmantas Kalendra, Anna Gagor, Daria Szewczyk, Adam Sieradzki, Miroslaw Maczka, Vytautas Samulionis, Aron Walsh, Robertas Grigalaitis, Juras Banys

*Corresponding author, e-mail: mantas.simenas@ff.vu.lt

Supplementary Note 1: Initial sample characterization

Photography of $MA_{1-x}DMA_xPbBr_3$ crystals used in this study is presented in Supplementary Figure 1.

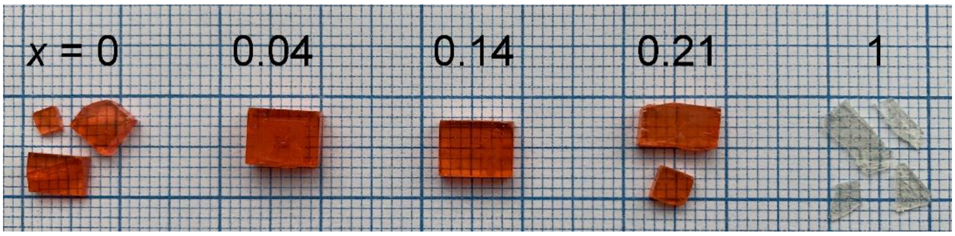

Supplementary Fig. 1 Crystals of mixed compounds. Examples of $MA_{1-x}DMA_xPbBr_3$ crystals used in this study.

The room temperature powder XRD experiments (Supplementary Figure 2) reveal that the crystal symmetry of all samples with $x \leq 0.21$ is cubic.[1] However, the XRD peaks shift to lower angles (inset in Supplementary Figure 2) indicating increase of the unit cell parameters due to incorporation of the bigger DMA cations.

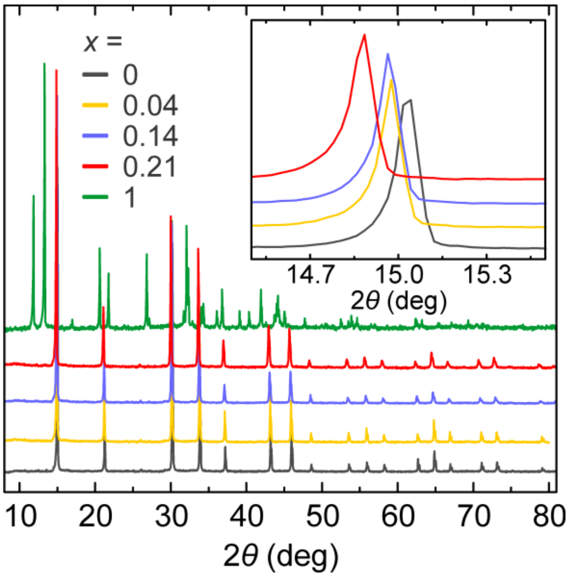

Supplementary Fig. 2 Powder XRD characterization. Room temperature powder XRD patterns of $MA_{1-x}DMA_xPbBr_3$ perovskites. The first peak is enlarged in the inset.

The Raman spectra of the prepared compounds are presented in Supplementary Figure 3. Upon increase of the DMA concentration, the new Raman bands appear due to the DMA cation vibrations. The most characteristic band is at 887 cm^{-1}, which corresponds to the C-N-C symmetric stretching.[2]

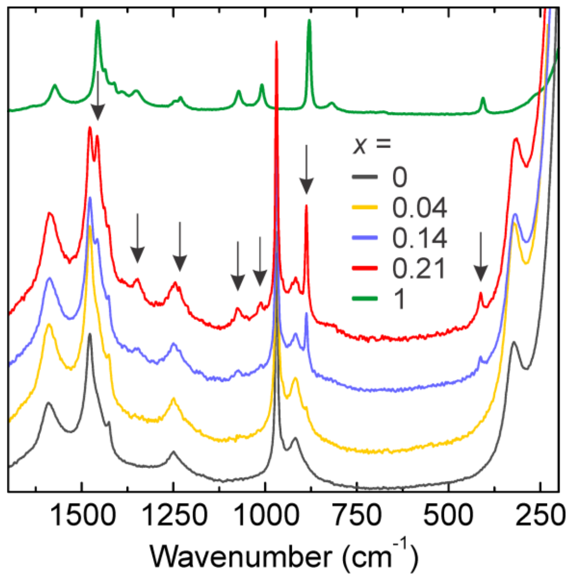

Supplementary Fig. 3 Raman spectroscopy characterization. Room temperature Raman spectra of $MA_{1-x}DMA_xPbBr_3$ perovskites. The arrows mark bands of the DMA cations in the mixed compounds.

The light absorption measurements were performed to investigate the change of the crystal bandgap upon addition of the DMA cations (Supplementary Figure 4a). The Kubelka-Munk analysis (Supplementary Figure 4b) of the absorption data revealed a small blueshift of the bandgap from about 2.22 eV (x = 0) to 2.27 eV (x = 0.21). The bandgap determined for $DMAPbBr_3$ compound is about 3 eV. Our results are in agreement with previous studies.[3,4]

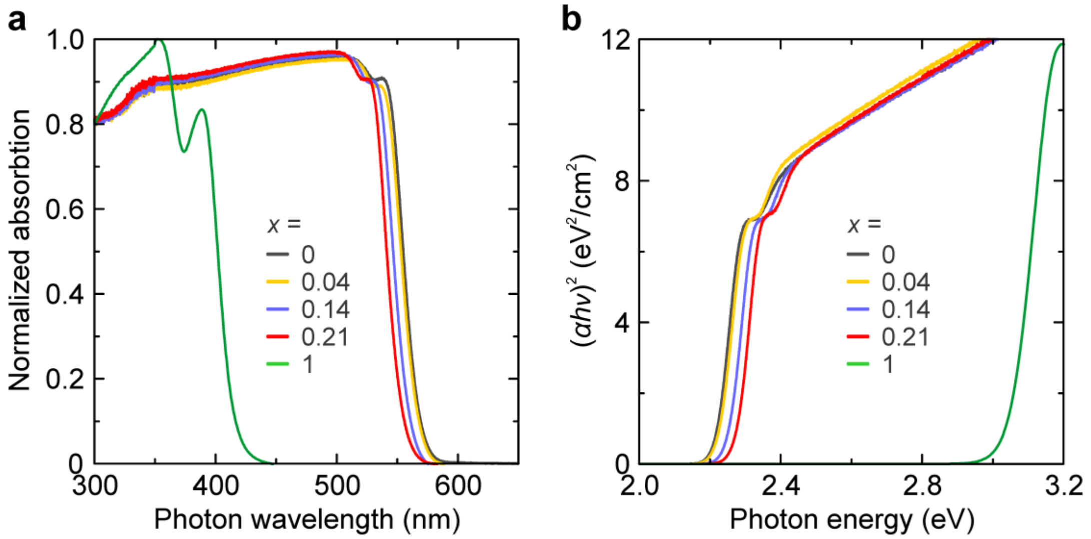

Supplementary Fig. 4 Light absorption characterization. a Light absorption spectra and **b** Kubelka-Munk plot of $MA_{1-x}DMA_xPbBr_3$ perovskites.

Supplementary Note 2: Additional experimental data

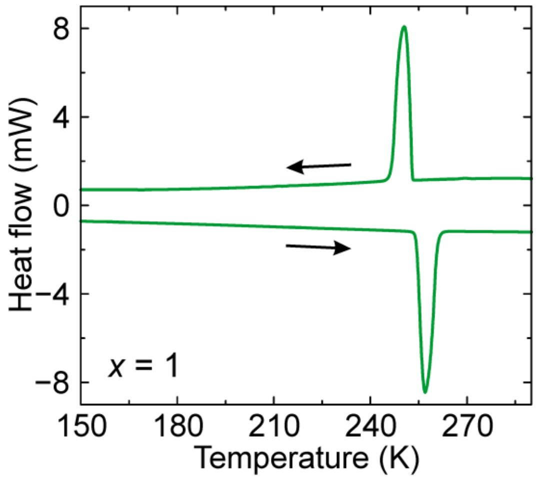

Supplementary Fig. 5 DSC data of $DMAPbBr_3$. DSC trace of $DMAPbBr_3$ obtained on cooling and heating.

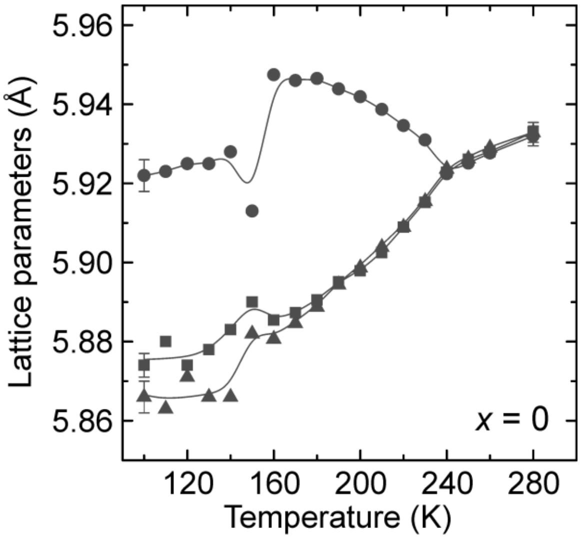

Supplementary Fig. 6 Single crystal XRD of $MAPbBr_3$. Temperature dependence of the crystal lattice parameters of $MAPbBr_3$ perovskite determined by the single crystal XRD. The curves are guides for eyes.

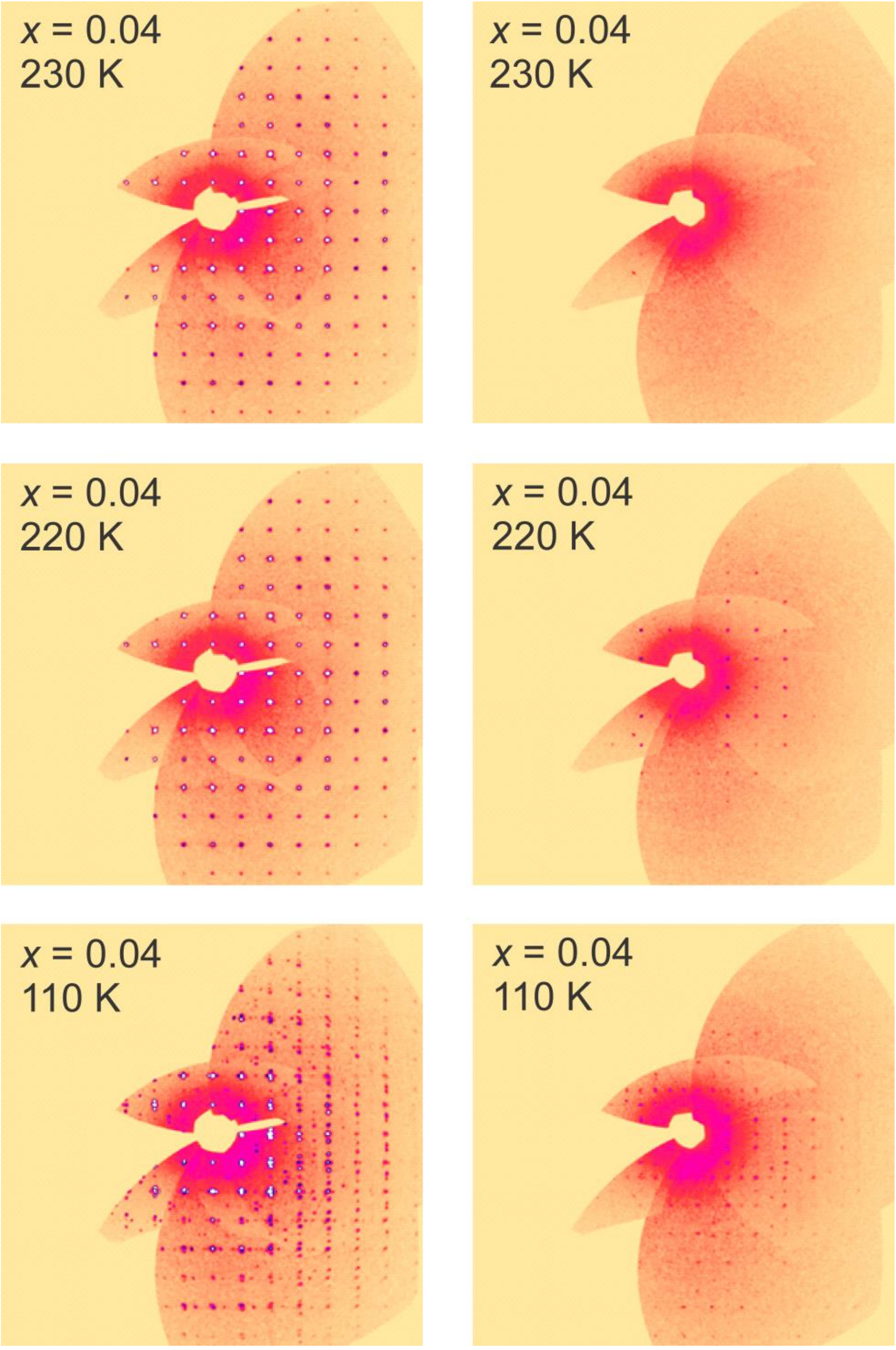

Supplementary Fig. 7 Reciprocal space reconstruction of the *x* = 0.04 compound at different temperatures. hk0 layers on the left, hk0.5 layers on the right. The Bragg peaks are characteristic of the cubic phase at 230 K. The lattice doubling gives additional intensities at hkn.5 layers (where n is an integer) at 220 K (tetragonal phase). Due to the formation of three structural domains in the tetragonal phase, the additional peaks are observed also at n.5kl and hn.5l layers. Further cooling decreases the symmetry to the orthorhombic, which results in a significant splitting of the diffraction peaks, since *m3m* to *mmm* symmetry reduction may result in six twin domains. hk0 and hk0.5 at 110 K are characteristic of the orthorhombic phase.

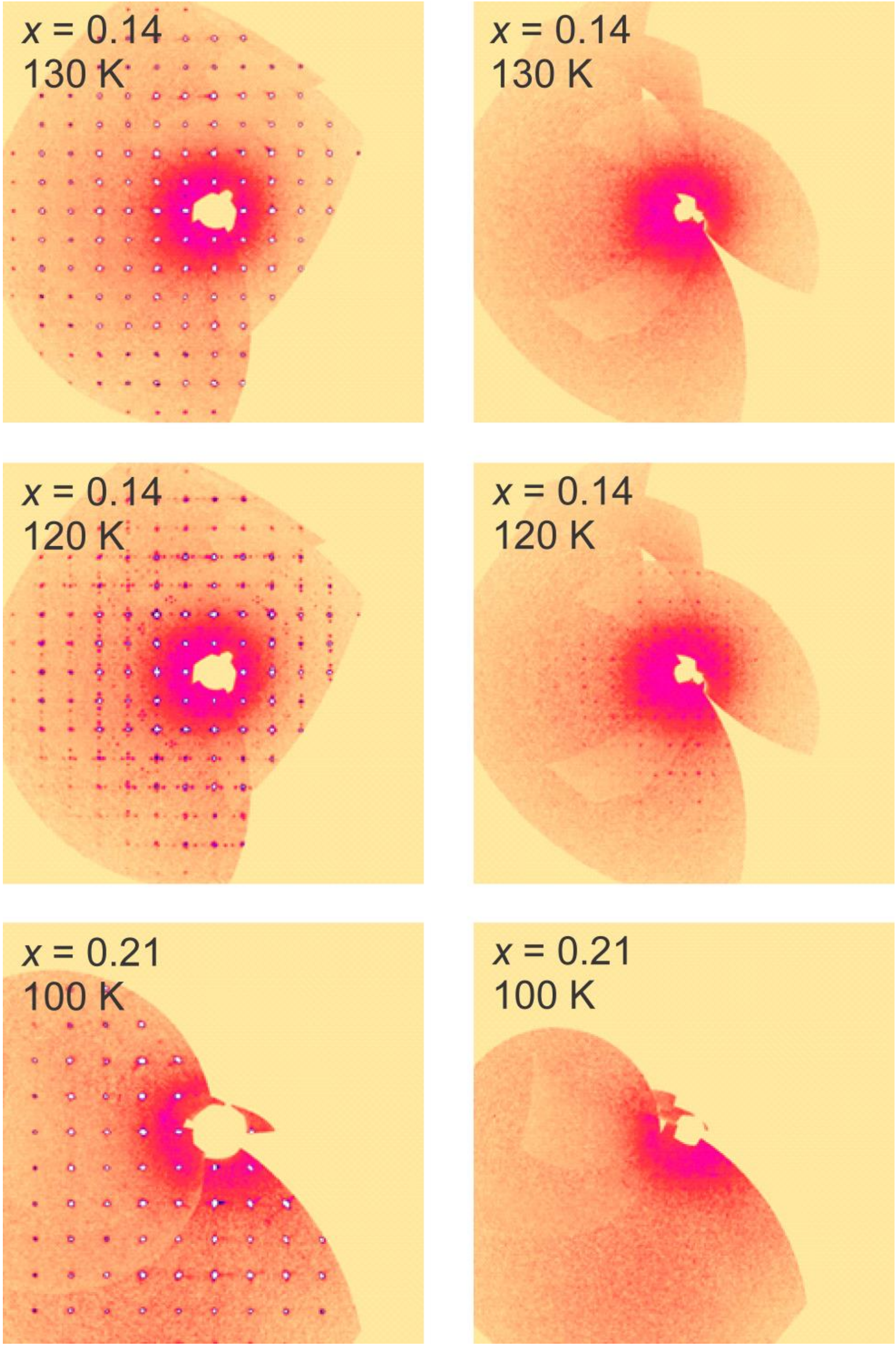

Supplementary Fig. 8 Reciprocal space reconstruction of the *x* = 0.14 and 0.21 compounds at different temperatures. hk0 layers on the left, hk0.5 layers on the right. For *x* = 0.14 the diffraction images are characteristic of the cubic phase down to 130 K. Below this temperature, a significant splitting of Bragg peaks resembles the orthorhombic phase. For *x* = 0.21 the diffraction images are characteristic of the cubic phase.

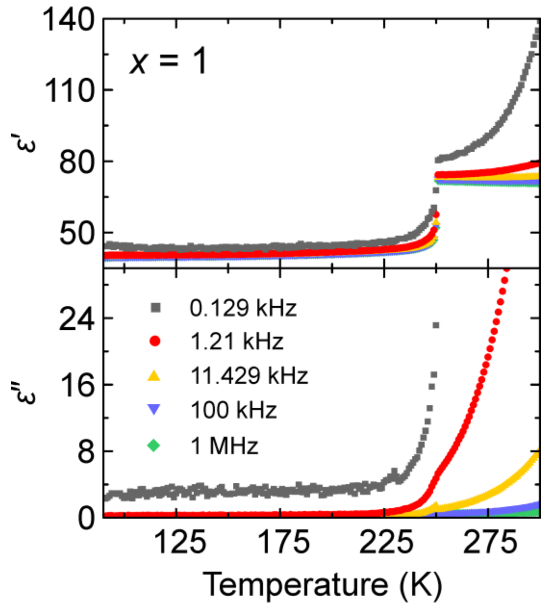

Supplementary Fig. 9 Dielectric properties of $DMAPbBr_3$. Temperature dependence of the complex dielectric permittivity of $DMAPbBr_3$ single crystal.

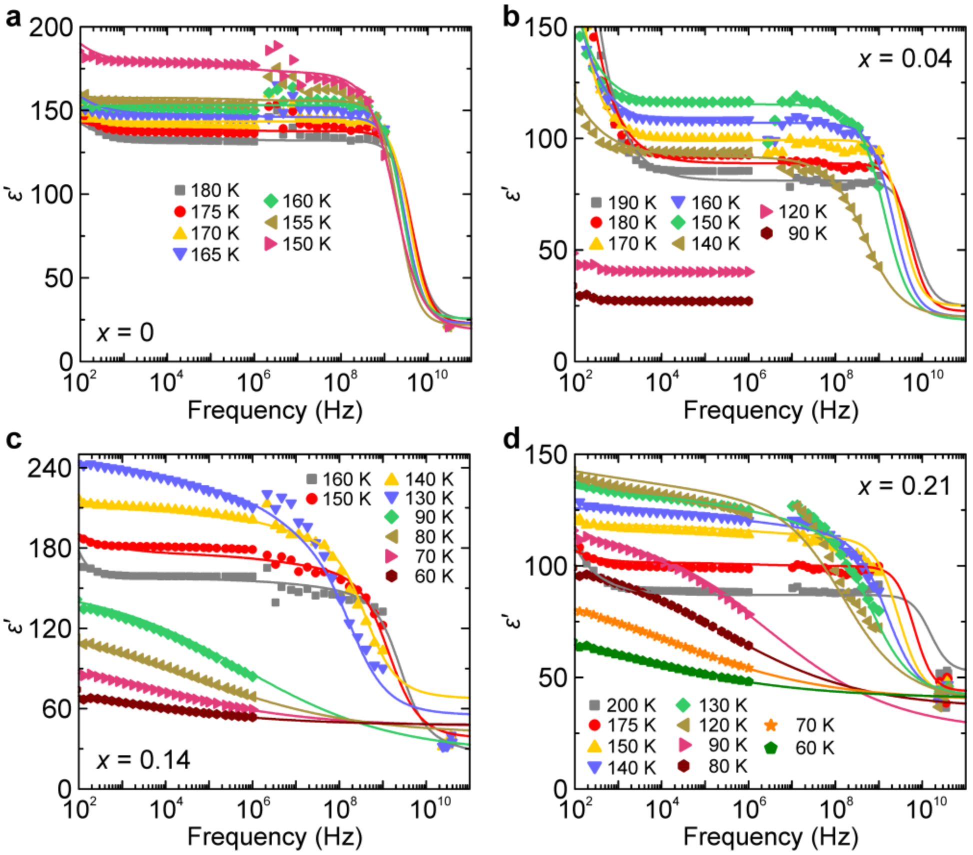

Supplementary Fig. 10 Frequency dependent dielectric permittivity of mixed $MA_{1-x}DMA_xPbBr_3$ perovskites. Frequency dependence of the real part of the complex dielectric permittivity at selected temperatures of **a** $x = 0$, **b** 0.04, **c** 0.14 and **d** 0.21 single crystal compounds. The solid curves are the best fits to Eq. 1.

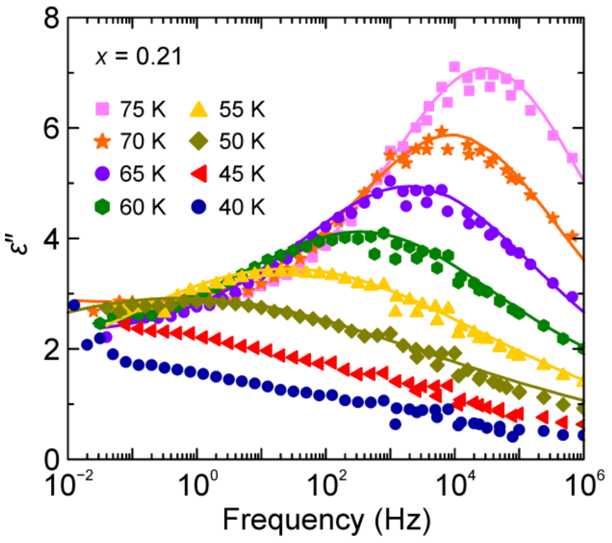

Supplementary Fig. 11 Low-frequency dielectric characterization of *x* = 0.21 compound. Low-frequency dependence of the imaginary part of the complex dielectric permittivity at selected temperatures of *x* = 0.21 single crystal compound. The curves are the best fits to Eq. 1.

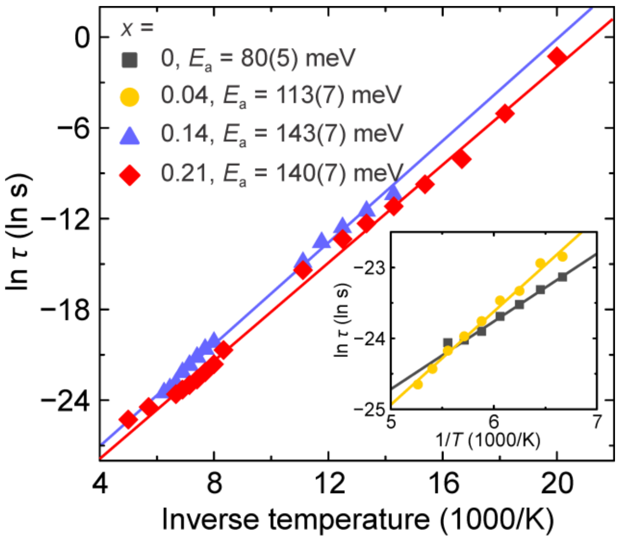

Supplementary Fig. 12 Arrhenius behavior of the mixed compounds. Inverse temperature dependence of the mean relaxation time of the *x* = 0, 0.04 (inset), 0.14 and 0.21 single crystal compounds measured by the broadband dielectric spectroscopy. The solid curves indicate fits to the Arrhenius equation.

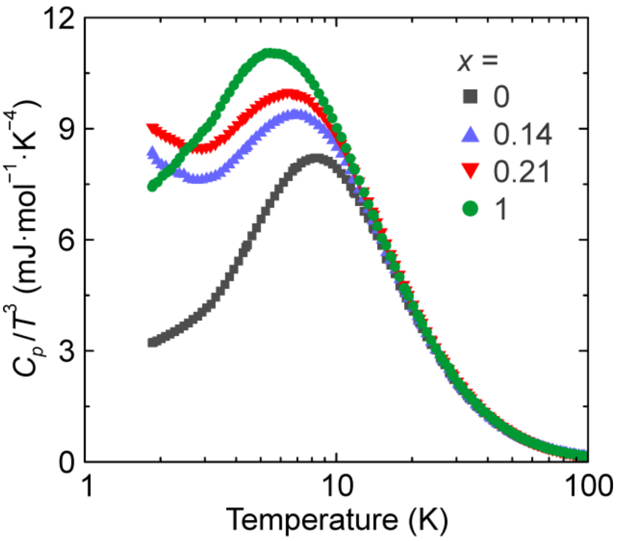

Supplementary Fig. 13 Heat capacity of mixed compounds. Temperature dependence of C_p/T^3 of the x = 0, 0.14, 0.21 and 1 compounds.

Supplementary Note 3: Additional computational data

The optimized lowest energy structure obtained by DFT calculations is presented in Supplementary Figure 14 (2 × 2× 2 supercell with one DMA cation corresponds to x = 0.125). The MA cations are approximately oriented along the <110> (or equivalent) direction. The DMA cation is situated in the center of the lead-bromine cuboid cavity with two amine protons pointing to the halogens and C-C bond directed along the <001> (or equivalent) direction. The dipole moment of the DMA molecules is orientated at approximately 45° with respect to the overall checkerboard arrangement created by the MA dipole moments. Such a configuration maximizes the H-bonding with the halogen atoms.

The lowest energy structure was found to deviate from the expected cubic structure. The lattice deformation, which is centered around the DMA cation, is such that the resultant crystal structure cannot be assigned to one of the 237 crystallographic space groups. As the lattice parameters are a = 11.85, b = 11.84, c = 11.77 Å, we will use the language of 'pseudo-tetragonal' to describe the relaxed structure. As XRD measurements involve spatial or temporal averaging, the measurements which suggest that the x = 0.14 and 0.21 samples remain cubic may be rationalized by a distribution in local lattice deformations. The direction of the lattice deformation is correlated to the orientation of the DMA molecule, and therefore a distribution of lattice deformations is to be expected from statistical thermodynamic considerations.

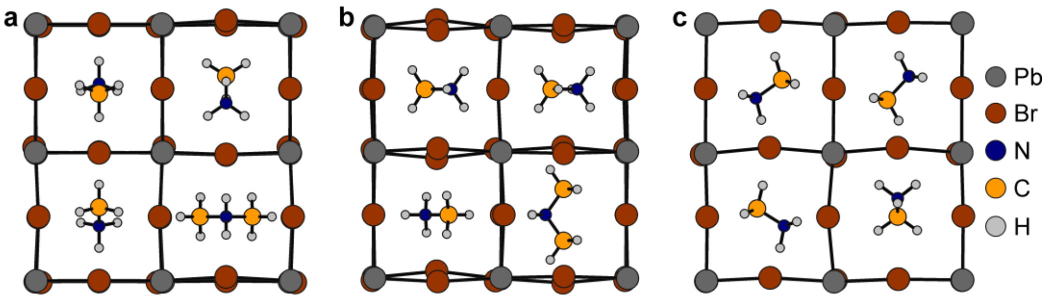

Supplementary Fig. 14 DFT calculations of the mixed compound. Lowest energy structure of $MA_{0.875}DMA_{0.125}PbBr_3$ obtained by DFT calculations, as seen from the **a** <100>, **b** <010>, and **c** <001> directions. For clarity, only a 2 × 2 × 1 slab containing DMA is presented.

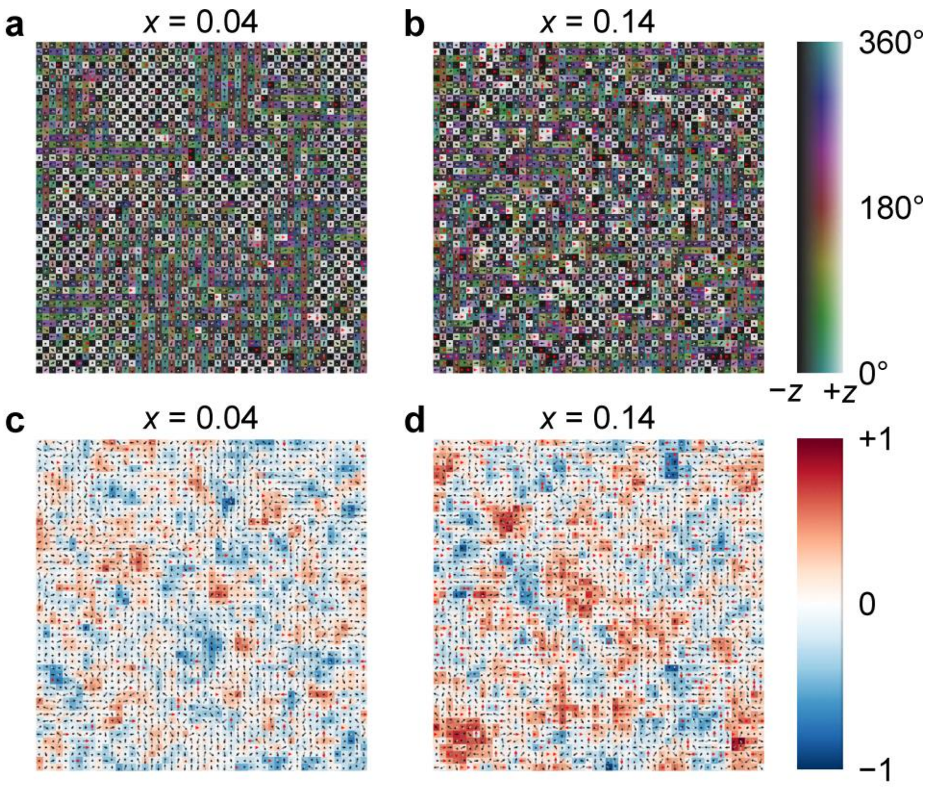

Supplementary Fig. 15 Monte Carlo simulations of mixed $MA_{1-x}DMA_xPbBr_3$ perovskites. a,b Snapshots of Monte Carlo simulations and **c,d** potential energy landscape for a two-dimensional slice of a three-dimensional periodic slab representing $MA_{1-x}DMA_xPbBr_3$ for **a,c** x = 0.04, and **b,d** x = 0.14. The orientations of the MA and DMA dipoles are represented by grey and red arrowheads, respectively. The MA dipole is allowed to freely rotate however, the orientation of the DMA dipole is fixed toward a <100> (or equivalent) lattice direction. The color bar in **a,b** represents the angle of the dipole with respect to the <100> direction; it is darkened towards <00-1> (-z) and lightened towards <001> (+z). The color bar in **c,d** shows the normalized electrostatic potential.

Supplementary References

(1) Wang, K.-H.; Li, L.-C.; Shellaiah, M.; Wen Sun, K. Structural and Photophysical Properties of Methylammonium Lead Tribromide ($MAPbBr_3$) Single Crystals. *Sci. Rep.* **7**, 13643 (2017).

(2) Maczka, M.; Ptak, M.; Macalik, L. Infrared and Raman Studies of Phase Transitions in Metal-Organic Frameworks of $[(CH_3)_2NH_2][M(HCOO)_3]$ with M=Zn, Fe. *Vib. Spectrosc.* **71**, 98–104 (2014).

(3) Anelli, C.; Chierotti, M. R.; Bordignon, S.; Quadrelli, P.; Marongiu, D.; Bongiovanni, G.; Malavasi, L. Investigation of Dimethylammonium Solubility in $MAPbBr_3$ Hybrid Perovskite: Synthesis, Crystal Structure, and Optical Properties. *Inorg. Chem.* **58**, 944–949 (2019).

(4) Garcia-Fernandez, A.; Juarez-Perez, E. J.; Bermudez-Garcia, J. M.; Llamas-Saiz, A. L.; Artiaga, R.; Lopez-Beceiro, J.; Senaris-Rodriguez, M. A.; Sanchez-Andujar, M.; Castro- Garcia, S. Hybrid Lead Halide $DMAPbX_3$ (X=Cl^- and Br^-) Perovskites with Multiple Functional Properties. *J. Mater. Chem. C* **7**, 10008–10018 (2019).
